# Perinatal predictors of clinical instability at birth in late-preterm and term infants

**DOI:** 10.1007/s00431-022-04684-5

**Published:** 2022-11-23

**Authors:** Georgia A. Santomartino, Douglas A. Blank, Alissa Heng, Anthony Woodward, Stefan C. Kane, Marta Thio, Graeme R. Polglase, Stuart B. Hooper, Peter G. Davis, Shiraz Badurdeen

**Affiliations:** 1 Newborn Research Centre, The Royal Women’s Hospital, 20 Flemington Rd, Parkville, VIC 3052 Australia; 2 The Ritchie Centre, Hudson Institute of Medical Research, 27-31 Wright St, Clayton, VIC Australia; 3 Faculty of Medicine, Nursing and Health Sciences, Monash University, 27 Rainforest Walk, Clayton, VIC Australia; 4 Division of Maternity Services, The Royal Women’s Hospital, 20 Flemington Rd, Parkville, VIC Australia; 5 Department of Maternal Fetal Medicine, The Royal Women’s Hospital, 20 Flemington Rd, Parkville, VIC Australia; 6 Department of Obstetrics and Gynaecology, The University of Melbourne, Parkville, VIC Australia; 7 Department of Obstetrics and Gynaecology, Monash University, Wellington Rd, Clayton, VIC Australia; 8 Department of Paediatrics, Monash University, Wellington Rd, Clayton, VIC Australia; 9 Monash Newborn, Monash Children’s Hospital, 246 Clayton Rd, Clayton, VIC Australia; 10 Clinical Sciences Research, Murdoch Children’s Research Institute, Flemington Rd, Parkville, VIC Australia

**Keywords:** Instability, Resuscitation, Neonatology, Birth

## Abstract

To identify characteristics associated with delivery room clinical instability in at-risk infants. Prospective cohort study. Two perinatal centres in Melbourne, Australia. Infants born at ≥ 35^+0^ weeks’ gestation with a first-line paediatric doctor requested to attend. Clinical instability defined as any one of heart rate < 100 beats per minute for ≥ 20 s in the first 10 min after birth, maximum fraction of inspired oxygen of ≥ 0.70 in the first 10 min after birth, 5-min Apgar score of < 7, intubated in the delivery room or admitted to the neonatal unit for respiratory support. Four hundred and seventy-three infants were included. The median (IQR) gestational age at birth was 39^+4^ (38^+4^—40^+4^) weeks. Eighty (17%) infants met the criteria for clinical instability. Independent risk factors for clinical instability were labour without oxytocin administration, presence of a medical pregnancy complication, difficult extraction at birth and unplanned caesarean section in labour. Decision tree analysis determined that infants at highest risk were those whose mothers did not receive oxytocin during labour (25% risk). Infants at lowest risk were those whose mothers received oxytocin during labour and did not have a medical pregnancy complication (7% risk).

*Conclusions*: We identified characteristics associated with clinical instability that may be useful in alerting less experienced clinicians to call for senior assistance early. The decision trees provide intuitive visual aids but require prospective validation.

**Table:** 

**What is Known:**
• *First-line clinicians attending at-risk births may need to call senior colleagues for assistance depending on the infant’s condition.* • *Delays in effectively supporting a compromised infant at birth is an important cause of neonatal morbidity and infant-mother separation.*
**What is New:**
• *This study identifies risk factors for delivery room clinical instability in at-risk infants born at* ≥ *35*^+*0*^* weeks’ gestation.* • *The decision trees presented provide intuitive visual tools to aid in determining the need for senior paediatric presence.*

**Supplementary Information:**

The online version contains supplementary material available at 10.1007/s00431-022-04684-5.

## Introduction

Many infants require assistance to successfully complete the transition from fetal to neonatal life [1]. Perinatal characteristics are widely used to identify infants at-risk, but the amount of assistance required can vary. In many settings, at-risk births are attended by a first-line paediatric doctor whose skill level can vary based partly on prior experience. First-line clinicians attending births often call for help from senior colleagues if the infant requires prolonged or advanced interventions.

Predicting which infants may benefit from a senior’s presence is challenging. Delays in recognising and/or effectively supporting a compromised infant increase the risk of neonatal morbidity and infant-mother separation [2, 3]. Accurate identification of infants at risk of needing more advanced stabilisation may inform hospital policies and ensure the best utilisation of staff. Less experienced clinicians may also feel better supported in the presence of a more senior colleague.

We hypothesised that among births attended by first-line paediatric clinicians, perinatal characteristics could help identify a subgroup of infants where an early call for senior assistance is appropriate. We use the term *clinical instability* to describe these instances based on physiological parameters and/or the level of resuscitation provided. Our aims were to:

1. Identify characteristics associated with increased risk of clinical instability in the delivery room for infants born at ≥ 35^+0^ weeks’ gestation.
2. Describe the probability of clinical instability in relation to these risk factors.

## Methods

We used data from infants concurrently recruited to a randomised controlled trial and prospective cohort study (ACTRN12618000621213) [4] at The Royal Women’s Hospital (RWH) and Monash Medical Centre (MMC) in Melbourne, Australia between 4 July 2018 and 18 May 2021. These are tertiary perinatal centres supporting births both for the local population as well as for high-risk pregnancies from a wide geographical region with similar referral criteria. Approval was obtained from each site’s Human Research Ethics Committee. Informed written parental consent was obtained for all participants, and deferred consent was obtained in emergency situations where prospective consent was not possible. This study is reported in accordance with the Strengthening the Reporting of Observational Studies in Epidemiology (STROBE) statement [5].

### Participants

Infants were eligible if they fulfilled all of the following inclusion criteria:

-  ≥ 35^+0^ weeks’ gestation at birth.
- Paediatric doctor requested by the obstetric or midwifery team to attend an at-risk birth, as determined by local hospital policy (online supplementary Fig. 1).
- Researcher present at the birth.

Infants were ineligible if any of the following exclusion criteria were fulfilled:

- Known congenital anomalies compromising cardiorespiratory transition
- High risk of obstetric complications requiring early cord clamping, including abnormal placentation, abruption, suspected uterine rupture and coagulopathy.
- Monochorionic twins and multiples > 2.

Infants assessed as requiring resuscitation within 1 min of birth were randomised to either physiologically based cord clamping, where resuscitation was commenced and effective respiratory support, if needed, was provided for at least 1 min before umbilical cord clamping, or standard care, where cord clamping occurred early, prior to resuscitation. Infants who were vigorous immediately after birth were not randomised and instead were included in the observational cohort study. Some infants who were initially vigorous went on to receive stabilisation in the delivery room. Infants in both the randomised and non-randomised study arms were included in the present analysis. The decision to provide resuscitation interventions and the type of support provided were at the discretion of the attending first-line doctor trained in the Australian and New Zealand Committee on Resuscitation Neonatal Resuscitation Guidelines [6].

Immediately after birth, a researcher dried the infant and placed three ECG chest leads and a preductal pulse oximeter to monitor the infant’s heart rate (HR) and oxygen saturation (SpO_2_). HR and SpO_2_ were displayed on a portable Intellivue X2 (Philips Healthcare, USA) or Infinity M540 (Dräger, Germany) monitor, visible to the clinician. A GoPro Hero Session (GoPro, USA) captured the monitor screen, T-piece manometer (NeoPuff™, Fisher&Paykel Healthcare, NZ), oxygen blender dial and an audio recording of the events after birth. The videos were downloaded for offline data extraction. HR and SpO_2_ data were manually extracted at 10-s intervals for 10 min after birth. Data points containing poor QRS or SpO_2_ waveforms were excluded. Data were extracted unblinded to the study outcome by four assessors (GS, AH, SB, DB), trained by two senior researchers (SB, DB) who also verified datapoints where HR and SpO_2_ were uncertain.

Clinical instability was defined as any of the following criteria, which the study authors defined a priori as suitable thresholds for senior escalation:

- HR < 100 beats per minute (bpm) for ≥ 20 s in the first 10 min after birth
- Maximum fraction of inspired oxygen (FiO_2_) of ≥ 0.70 in the first 10 min after birth
- 5-min Apgar score of < 7
- Intubation in the delivery room
- Admission to the neonatal unit for respiratory support

Controls were infants who displayed none of the above clinical instability criteria.

Maternal and infant demographic details, perinatal characteristics, resuscitation interventions and clinical outcomes were entered by the study researcher into the REDCap electronic study database immediately following the birth [7].

### Analysis

Recruitment for this study continued until the target sample size of 120 infants was reached for the randomised trial. There was no prespecified sample size for the prospective cohort arm of the study.

For the comparative analysis, we chose perinatal variables that might predict clinical instability based on clinical experience and previous studies [8–11]. We chose variables that are generally known by the time of birth so that help from a senior doctor might be requested in a timely manner.

Analyses were performed using IBM SPSS Statistics version 27 and *R* V.3.6.2 (R Foundation, Vienna, Austria). For categorical variables, comparison was performed using a χ^2^ test or Fisher’s Exact Test as appropriate. Continuous variables were compared using a two-sided *t*-test for normally distributed variables and a Mann–Whitney *U* test for skewed variables. We report risk ratios and their 95% CIs, and statistically significant differences were defined as those with *p* < 0.05. To simplify the number of risk factors and facilitate clinical applicability, we performed an adjusted analysis using backward stepwise log-binomial regression retaining variables with *p* < 0.1. We report risk ratios and their respective 95% CIs for variables in the final model with *p* < 0.05.

#### Decision tree analysis

In keeping with our aim to describe the probability of delivery room clinical instability in relation to the identified risk factors, we created conditional inference decision trees [12]. Decision trees have similar modelling ability to logistic regression with the advantage of generating an intuitive visual tool for clinical decision-making [13–15]. In this analysis, the study population is recursively split into subgroups based on the predictive variables that are most strongly related to the outcome. Using a Bonferroni-adjusted *p* value, the algorithm chooses which variables to split, their discriminatory value and the order in which the splitting occurs. Outcome discrimination can thus be maximised at each step by accounting for the relationships between variables while limiting both overfitting and biased variable selection [13]. We used the *partykit* package in *R* with default settings of the ‘ctree’ function. For the subgroup of infants born by vaginal birth, there was no Bonferroni-adjusted *p* value < 0.05 to generate any splits. We therefore used the chi square value for variable selection to generate an interpretable tree.

## Results

**Fig. 1:**
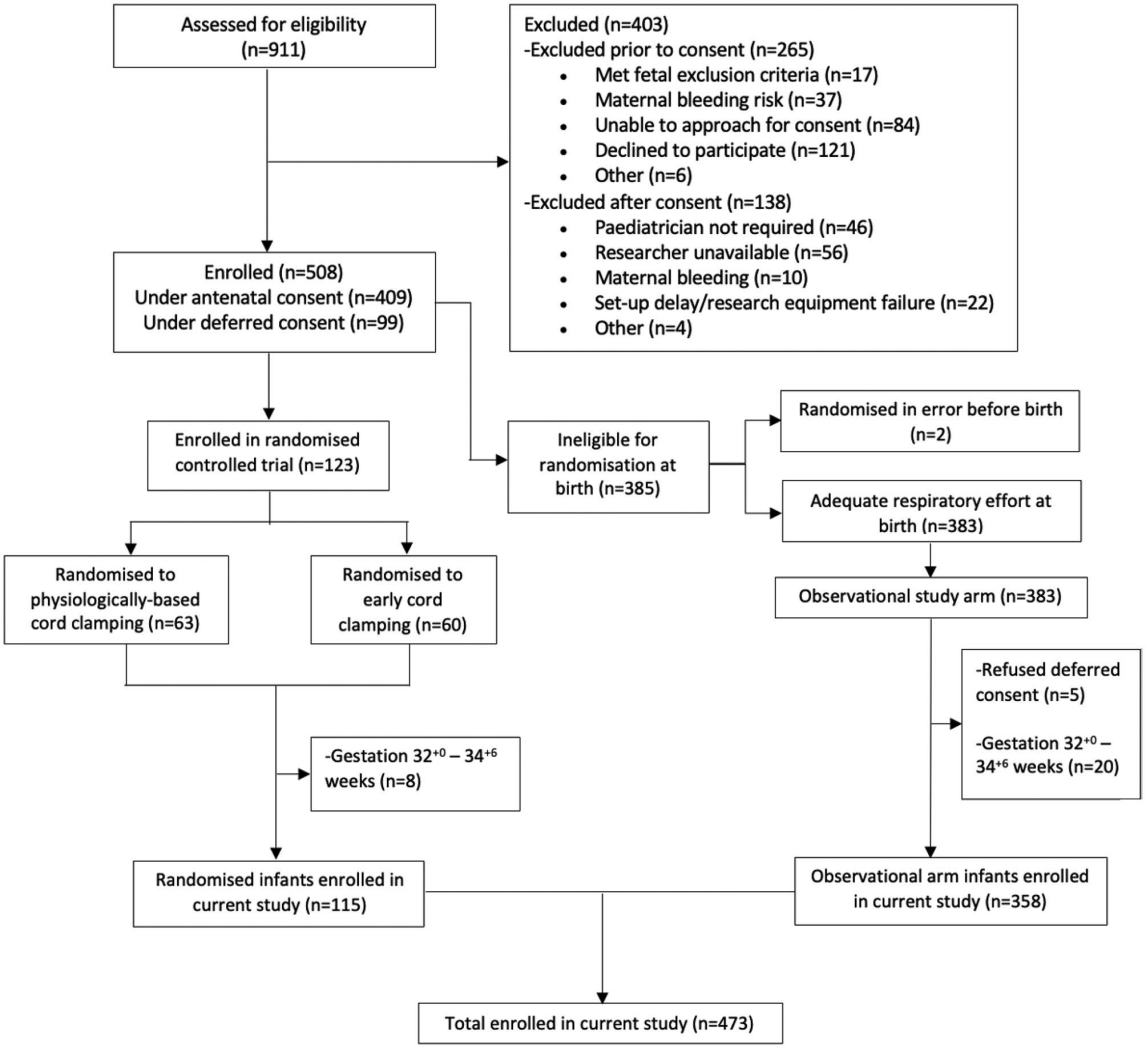
Diagram showing participant recruitment to the current study from the randomised controlled trial and prospective cohort study

A total of 473 infants were included in this study; 115 (24%) from the randomised controlled trial and 358 (76%) from the prospective cohort study (Fig. 1). The median (IQR) gestational age at birth for all infants was 39^+4^ (38^+4^–40^+4^) weeks (Table 1). Common reasons for paediatric attendance and therefore study inclusion were instrumental birth (41%), unplanned caesarean Sect. (33%) and meconium-stained amniotic fluid (27%). Approximately half the unplanned caesarean sections were in mothers with established labour (51%). For the majority of births, the lead accoucher was an obstetric registrar doctor (80%).

**Table 1:** Participant characteristics and clinical outcomes

	**All infants** ***N* = 473**	**Clinical instability** ***N* = 80**	**No clinical instability** ***N* = 393**	***p* value**
**Participant characteristics**				
Hospital at birth, n (%)^a^				
- Royal Women’s Hospital	414(88)	73(91)	341(87)	-
- Monash Medical Centre	59(12)	7(9)	52(13)	
Maternal age (years)^b^	33(4) *N* = 472^c^	32(4)	33(4) *N* = 392	-
Primiparity, n (%)	357(79) *N* = 451	63(83) *N* = 76	294(78) *N* = 375	-
Gestational age (weeks^+days^)	39^+4^ (38^+4^–40^+4^)	39^+3^ (37^+6^–40^+1^)	39^+4^ (37^+5^–40^+4^)	-
Birth weight (kg)	3.42(3.08–3.74) *N* = 472	3.39(3.01–3.66)	3.42(3.09–3.77) *N* = 392	-
Female sex, n (%)	231(49)	45(56)	186(47)	-
Infant from twin pregnancy, n (%)	6(1)	3(4)	3(1)	-
Accoucher, n (%)				
- Midwife	37(8)	2(3)	35(9)	
- Junior registrar	189(41)	35(47)	154(40)	
- Senior registrar	180(39)	29(39)	151(39)	
- Consultant	51(11) *N* = 457	8(11) *N* = 74	43(11) *N* = 383	
Strongest analgesia/anaesthesia, n (%)				
- None or Nitrous oxide	36(8)	3(4)	33(8)	
- Opiate (IV or IM)	4(1)	2(3)	2(1)	
- Spinal or epidural	428(90)	73(91)	355(90)	
- General anaesthetic	5(1)	2(3)	3(1)	
Reason for paediatric attendance, n (%)				
- Preterm, 35^+0^ –36^+6^ weeks’ gestation	39(8)	12(15)	27(7)	
- Fetal growth restriction	27(6)	4(5)	23(6)	
- Meconium-stained amniotic fluid	128(27)	16(20)	112(28)	
- Abnormal CTG	245(52)	30(38)	215(55)	
- Breech/transverse lie/unstable lie	84(18)	24(30)	60(15)	
- Instrumental birth	192(41)	20(25)	172(44)	
- Unplanned caesarean section	155(33)	35(44)	120(31)	
- Other	11(2)	1(1)	10(3)	
Timing of umbilical cord clamping, n (%)				
- Early cord clamping (0–60 s)	40(9)	14(18)	40(10)	
- Delayed/deferred cord clamping (> 60 s)	430(91)	66(83)	350(90)	
**Outcomes**				
Clinical instability criteria, n (%)^d^				
- Admitted to the neonatal unit for respiratory support	29(6)	29(36)	-	
- Heart rate < 100 bpm for ≥ 20 s	45(10)	45(56)	N/A	
- Maximum FiO_2_ ≥ 0.70	30(6)	30(38)	-	
- 5-min Apgar score of < 7	2(0)	2(3)	-	
- Intubated	1(0)	1(1)	-	
Respiratory support provided, n (%)				
- None	361(76)	19(24)	342(87)	<0.001^e^
- Any PPV	45(10)	25(31)	20(5)	<0.001
- CPAP + / − O_2_	60(13)	33(41)	27(7)	<0.001
- O_2_ alone	7(1)	3(4)	4(1)	0.098
Apgar score				
- 1 min	9(8–9) *N* = 472	8(5–9)	9(8–9) *N* = 392	< 0.001
- 5 min	9(9–9) *N* = 472	9(8–9)	9(9–9) *N* = 392	< 0.001
Time from birth to first cry (s)	20(5–35) *N* = 465	32(9–60) *N* = 79	18(4–32) *N* = 386	< 0.001
Admitted to the neonatal unit, n (%)	66(14)	38(48)	28(7)	< 0.001
Primary reason for admission, n (%)				
- Respiratory support	29(6)	29(36)	0(0)	< 0.001
- Prematurity or low birth weight alone	9(2)	2(3)	7(2)	
- Low glucose	15(3)	4(5)	11(3)	
- Other	13(3) *N* = 66	3(4) *N* = 38	10(3) *N* = 28	

^a^Categorical variables are presented as n (%)

^b^Continuous variables are presented as median (IQR) except for maternal age, which was normally distributed and is presented as mean (SD)

^c^Where N of a variable does not equal N of the column due to missing values, N of the variable is stated in the cell

^d^Infants can satisfy more than one clinical instability criterion

^e^*P* values represent univariable comparisons between cases of clinical instability versus controls for important clinical outcomes

*IV* intravenous, *IM* intramuscular, *CTG* cardiotocography, *bpm* beats per minute, *s* seconds, *FiO*_*2*_ fraction of inspired oxygen, *PPV* positive pressure ventilation, *CPAP* continuous positive airway pressure, *O*_*2*_ oxygen, *N/A* not applicable

Eighty (17%) infants met the criteria for clinical instability (Table 1). Among these cases, 56% had HR < 100 bpm for ≥ 20 s, 38% had an FiO_2_ ≥ 0.70, 3% had a 5 min Apgar score < 7, 1% were intubated and 36% were admitted for respiratory support.

Table 2 describes the unadjusted and adjusted comparisons for risk factors. In the adjusted comparisons, oxytocin was found to be protective; infants whose mother received oxytocin during labour had approximately one-third the risk of clinical instability compared to infants whose mother did not. The risk of clinical instability was approximately doubled among infants whose mother had a medical pregnancy complication, infants who had a difficult extraction at birth, and infants born via unplanned caesarean section in labour, compared to infants who did not have these risk factors.

**Table 2:** Univariable and multivariable analyses of characteristics associated with clinical instability in the delivery room

	**Clinical instability** **(*N* = 80), n (%)**	**No clinical instability (*N* = 393), n (%)**	**Crude risk ratio (95% confidence interval)**	***p* value**	**Adjusted risk ratio (95% confidence interval)**	***p* value**
Any medical complication of pregnancy^a,b^	35(44)	95(24)	2.04(1.38–3.02)	< 0.001	2.18(1.46–3.08)	< 0.001
Fetal compromise						
- Reduced movements	5(6)	23(6)	1.06(0.47–2.41)	0.799^c^		
- Meconium-stained amniotic fluid	16(20)	112(28)	0.67(0.41–1.12)	0.119		
- Abnormal CTG^d^	30(38)	215(55)	0.56(0.37–0.85)	0.005		
- Severely abnormal CTG^d,e^	4(5)	14(4)	1.33(0.55–3.23)	0.523^c^		
- Fetal growth restriction	4(5)	23(6)	0.87(0.34–2.20)	1.000^c^		
Spontaneous labour onset	21(26)	126(32)	0.79(0.50–1.25)	0.306		
Oxytocin during labour	28(35)	235(60)	0.43(0.28–0.66)	< 0.001	0.38(0.22–0.63)	< 0.001
Breech/transverse lie/unstable lie^f^	24(30)	60(15)	1.99(1.31–3.01)	0.002		
Macrosomia	3(4)	11(3)	1.28(0.46–3.56)	0.715^c^		
Labour complications						
- Prolonged rupture of membranes	4(5)	18(5)	1.08(0.43–2.68)	0.776^c^		
- Failure to progress	6(8)	57(15)	0.53(0.24–1.16)	0.093		
- Prolonged second stage	11(14)	100(25)	0.52(0.29–0.95)	0.024		
- Difficult extraction^g^	39(49)	100(25)	2.22(1.50–3.28)	< 0.001	1.97(1.29–2.86)	0.002
Type of birth						
- Unassisted vaginal	4(5)	44(11)	0.47(0.18–1.22)	0.094		
- Instrumental	20(25)	172(44)	0.49(0.30–0.78)	0.002		
- Planned CS^f^	21(26)	57(15)	1.80(1.17–2.78)	0.010		
- Unplanned CS in labour	16(20)	63(16)	1.25(0.76–2.04)	0.386	2.03(1.19–3.06)	0.010
- Unplanned CS not in labour	19(24)	57(15)	1.63(1.04–2.56)	0.040		
Gestation at birth	-	-	0.85(0.74–0.97)	0.036		

^a^Includes hypertensive disorders of pregnancy (*n* = 38), antepartum haemorrhage (*n* = 10), sepsis or chorioamnionitis (*n* = 2), diabetes mellitus or gestational diabetes (*n* = 55), multiple pregnancy (*n* = 8), oligohydramnios (*n* = 16), polyhydramnios (*n* = 4) and placenta praevia (*n* = 1). Some women had more than one medical complication of pregnancy

^b^Data was unavailable for 2 infants for ‘any medical complication of pregnancy’

^c^Fisher’s Exact Test

^d^3 infants met criteria for both abnormal and severely abnormal CTG

^e^Severely abnormal CTG was defined as prolonged or profound fetal bradycardia resulting in emergency delivery

^f^63 (81%) planned caesareans were for breech/transverse lie/unstable lie

^g^Difficult extraction at caesarean section was defined as a prolonged delay between uterine entry and delivery of the fetus, and at vaginal births as prolonged requirement for instrumentation to complete delivery of the head and shoulders. This was determined by the attending researcher

*CTG* cardiotocography, *CS* caesarean section

**Fig. 2:**
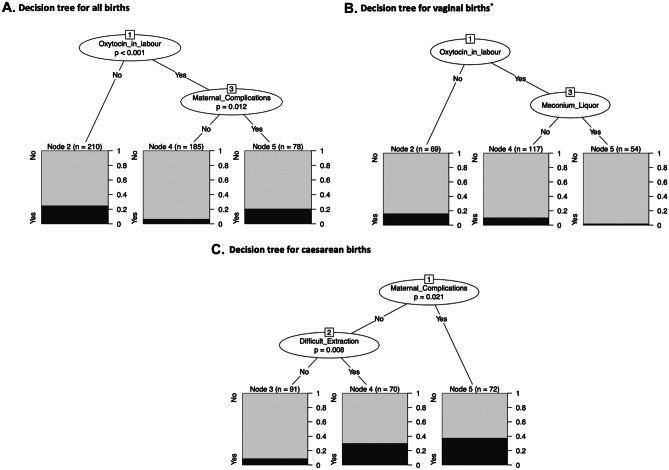
Decision trees depicting the most important risk factors in determining the likelihood of neonatal clinical instability at birth. **A** Decision tree for all studied births. Oxytocin during labour followed by maternal complications were the two most important predictive factors for neonatal clinical instability. **B** Decision tree for vaginal births. Oxytocin during labour followed by meconium liquor were the most important predictive factors in infants born vaginally. **C** Decision tree for caesarean births. Maternal complications followed by difficult extraction of the infant were the most important predictors in infants born via caesarean section. For all decision trees, the probability of showing signs of clinical instability at the terminal nodes is shaded in black. *For infants born vaginally, there was no Bonferroni-adjusted *p* value < 0.05 to generate any splits. As a result, we used the chi square value for variable selection to generate an interpretable tree; a method which is prone to overfitting

The decision trees in Fig. 2 provide flow charts that account for the overlap in risk factors. Infants at highest risk were those whose mothers did not receive oxytocin in labour (52 of 210, 25%) and those whose mothers did receive oxytocin in labour but had a medical complication of pregnancy (16 of 78, 21%) (Fig. 2A). Infants of mothers who received oxytocin in labour and did not have medical complications in pregnancy were at lowest risk (12 of 185, 7%).

The overall clinical instability risk in infants born vaginally was lower than that for infants born by caesarean birth (10% vs 24%, Table 2). Oxytocin during labour remained the most discriminatory factor of clinical instability risk (Fig. 2B). Infants at lowest risk of clinical instability were those whose mother received oxytocin during labour and had meconium-stained liquor (1 of 54, 2%). For caesarean births, infants of mothers with medical complications were at greatest risk (27 of 72, 38%, Fig. 2C). The absence of maternal medical complications and difficult extraction at birth identified a subgroup of infants at low risk for clinical instability (8 of 91, 9%).

## Discussion

Among births routinely attended by first-line paediatric clinicians, risk factors for clinical instability included absence of oxytocin administration in labour, any medical complication of pregnancy, difficult extraction at birth and unplanned caesarean section in labour. One in three infants with clinical instability were admitted to the neonatal unit for respiratory distress, indicating a strong association with clinically important outcomes.

Most previous studies describing risk factors for delivery room respiratory support and/or advanced resuscitation used all births as the denominator, rather than the subset of births routinely attended by first-line clinicians [9–11]. As such, these studies are mostly informative for guiding when a clinician should attend a birth. Only two studies described risk factors among at-risk births attended by a newborn resuscitation team. Yangthara et al. [16] and Aziz et al. [8] also found that maternal conditions in pregnancy, including hypertensive disorders, placental disorders, diabetes, infection and oligohydramnios, were associated with increased risk of receiving respiratory support and/or advanced resuscitation.

Previous studies did not include oxytocin administration during labour as a covariate. We included this variable as oxytocin stimulates uterine contraction and may compromise fetal oxygenation [17]. Sousa et al. found that induction of labour was not significantly associated with the need for positive-pressure ventilation at birth [9]. We found that oxytocin administration in labour was strongly associated with reduced risk of clinical instability. In our population, 71% of infants whose mothers received oxytocin during labour had an abnormal CTG and 35% were born via caesarean section. Women receiving oxytocin in actively managed labour are observed closely with CTG monitoring. Signs of fetal compromise may therefore be recognised sooner, prompting early delivery. Additionally, contractions in labour cause an increase in airway pressure and lung liquid clearance from the trachea, likely explaining the common occurrence of liquid loss from an infant’s nose and mouth following a vaginal birth [18]. As oxytocin increases the strength, duration and frequency of uterine contractions [17], this effect may be more pronounced. Further, labour exposes a fetus to hypoxia [19], so a reduction in the time spent in labour via oxytocin administration may reduce hypoxic exposure and aid neonatal respiratory transition.

The risk of clinical instability at instrumentally assisted vaginal births was similar to that at unassisted vaginal births (10% vs 8%). This is likely to reflect local obstetric practice, where there was a low threshold to provide assistance at monitored births. Instrumental births were not significantly associated with clinical instability in the multivariable analysis, possibly due to overlap with oxytocin administration during labour that had a greater protective effect. Unplanned caesarean section in labour was, however, associated with increased clinical instability risk in the multivariable analysis.

Difficult extraction of the fetus was also independently associated with clinical instability and featured in the decision tree for caesarean births. This variable was not included in previous studies. We included this variable based on clinical experience of instances where the first-line clinician calls for senior support, or is advised to by obstetric colleagues, in anticipation of poor neonatal condition at birth. Difficult extraction was determined to have occurred by the attending researcher if there was prolonged delay between uterine entry and delivery of the fetus at caesarean section, or prolonged requirement for instrumentation/manoeuvres to complete delivery of the head and shoulders at vaginal births. As this was a subjective assessment without pre-defined criteria, this variable may be unreliable. Clinical utility of the variable may also be undermined by the fact that it can only be known at the time of birth and is generally assessed in retrospect. However, we felt that inclusion of the variable was justified as it provided additional characterisation of the circumstances around each birth not otherwise represented by the other study variables. Working towards more objective measures of a difficult extraction at birth would be useful.

Infants born through meconium-stained amniotic fluid have increased likelihood of experiencing respiratory distress [20]. However, among the subset of vaginal births studied, decision tree analysis found that infants born with meconium-stained amniotic fluid following labour augmented with oxytocin were at lowest risk of clinical instability. Our findings may be partially explained by expedited delivery of fetuses showing signs of distress. Yangthara et al. [16] and Aziz et al. [8] both found meconium-stained amniotic fluid to be a risk factor, but direct tracheal suction and/or intubation was routine in their cohorts. The differences in findings may therefore reflect both changes in resuscitation practice and improvements in obstetric practice [21].

Strengths of this study include the inclusion of variables previously omitted by other studies that gave added granularity to the dataset, and the prospective recruitment of at-risk births from 2 sites with contemporary obstetric and neonatal practice. A greater number of infants were born at The RWH compared to MMC (414 vs 59 infants) due to differing researcher availability at each hospital. However, the proportion of infants with clinical instability at each site was similar (18% at The RWH vs 12% at MMC), reflecting the similar nature of births attended at both sites. These are tertiary perinatal centres. Further to the limitations highlighted above, most infants were recruited in-hours, so our population may not be representative of infants born overnight who may be delivered in more emergent situations. Time of birth is likely to be one of several other potential predictive factors that may further enrich the ability to accurately predict episodes of clinical instability. Our results, including the decision trees, reflect the local context and recruitment strategy. Therefore, prospective validation of our findings in a variety of settings is warranted prior to clinical application.

This study identifies characteristics associated with increased clinical instability risk among the large number of at-risk births typically attended by first-line paediatric doctors. We use decision trees to provide an intuitive visual tool for clinical decision-making. Following prospective validation, these models may enable clinicians to quickly identify when to call for a senior colleague, ensuring appropriately skilled staff are present for infants at increased risk of clinical instability and to provide better support to less experienced colleagues.

## Supplementary Information

Below is the link to the electronic supplementary material.

Supplementary file1 (JPG 503 KB)

## Data Availability

Data are available upon reasonable request. Sharing of data will be considered for specific research projects. Requests should be sent to the corresponding author.
