# Supplementary figures and images for: Perinatal predictors of clinical instability at birth in late-preterm and term infants
Source: Eur J Pediatr. 2022 Nov 23;182(3):987–95. doi: 10.1007/s00431-022-04684-5 (PMC10023598; doi:10.1007/s00431-022-04684-5)

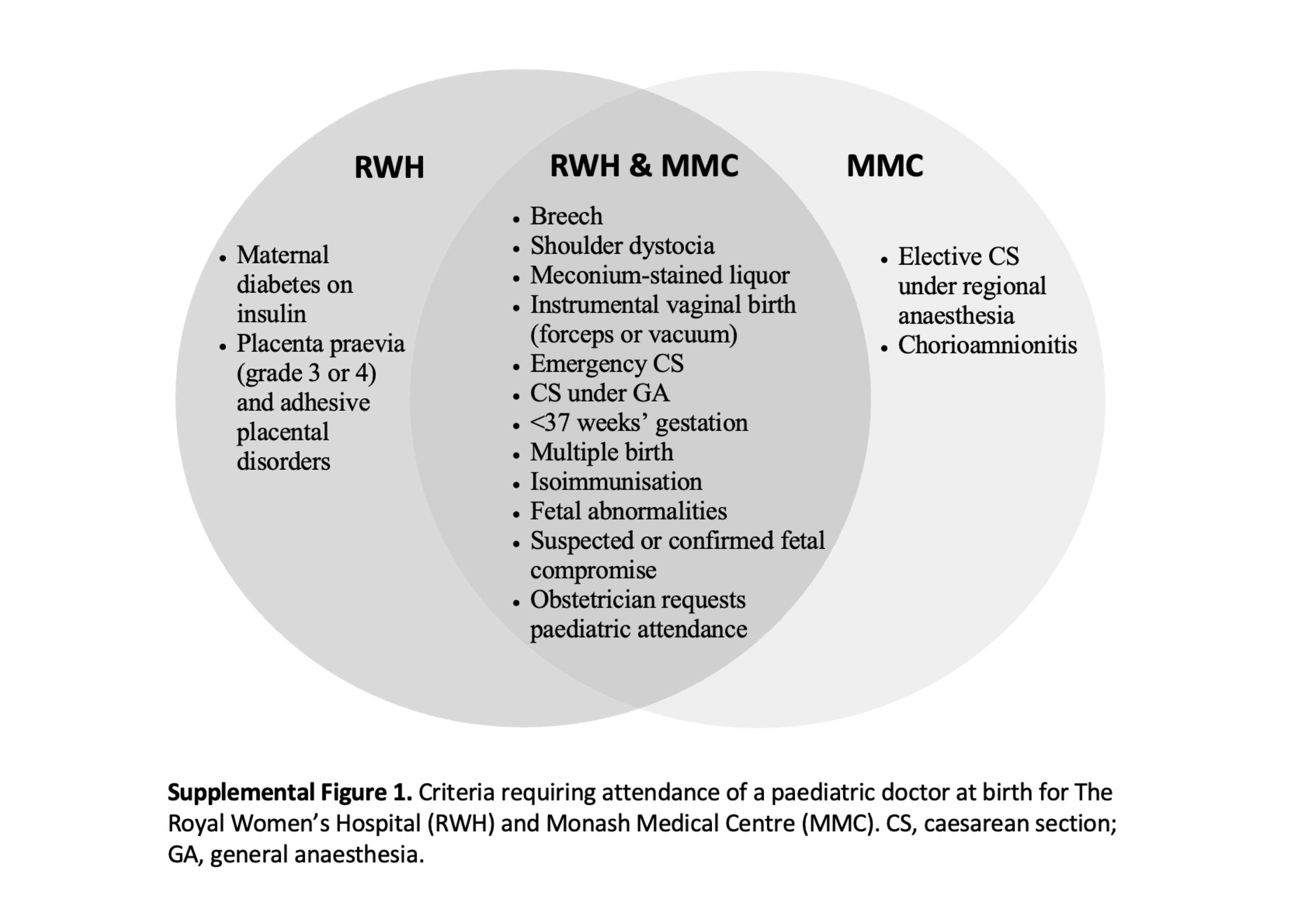

Supplement: Supplementary file 1 — Supplementary file1 (JPG 503 KB) [file 431_2022_4684_MOESM1_ESM.jpg]
